# Ultrasound microbubble-mediated delivery of the siRNAs targeting MDR1 reduces drug resistance of yolk sac carcinoma L2 cells

**DOI:** 10.1186/1756-9966-30-104

**Published:** 2011-10-28

**Authors:** Yun He, Yang Bi, Yi Hua, Dongyao Liu, Sheng Wen, Qiang Wang, Mingyong Li, Jing Zhu, Tao Lin, Dawei He, Xuliang Li, Zhigang Wang, Guanghui Wei

**Affiliations:** 1 Department of Urology, The Children's Hospital of Chongqing Medical University, Chongqing, People's Republic of China; 2 Key Laboratory of Developmental Diseases in Childhood, Chongqing Medical University, Ministry of Education, Chongqing, People's Republic of China; 3 Institute of Ultrasound Image, the Second Affiliated Hospital of Chongqing Medical University, Chongqing, People's Republic of China

**Keywords:** Yolk sac carcinoma, Ultrasound therapy, RNA interference, Multiple drug resistance gene, Transfection

## Abstract

**Background:**

MDR1 gene encoding P-glycoprotein is an ATP-dependent drug efflux transporter and related to drug resistance of yolk sac carcinoma. Ultrasound microbubble-mediated delivery has been used as a novel and effective gene delivery method. We hypothesize that small interfering RNA (siRNA) targeting MDR1 gene (siMDR1) delivery with microbubble and ultrasound can down-regulate MDR1 expression and improve responsiveness to chemotherapeutic drugs for yolk sac carcinoma *in vitro*.

**Methods:**

Retroviral knockdown vector pSEB-siMDR1s containing specific siRNA sites targeting rat MDR1 coding region were constructed and sequence verified. The resultant pSEB-siMDR1 plasmids DNA were encapsulated with lipid microbubble and the DNA release were triggered by ultrasound when added to culture cells. GFP positive cells were counted by flow cytometry to determine transfection efficiency. Quantitative real-time PCR and western blot were performed to determine the mRNA and protein expression of MDR1. P-glycoprotein function and drug sensitivity were analyzed by Daunorubicin accumulation and MTT assays.

**Results:**

Transfection efficiency of pSEB-siMDR1 DNA was significantly increased by ultrasound microbubble-mediated delivery in rat yolk sac carcinoma L2 (L2-RYC) cells. Ultrasound microbubble-mediated siMDR1s delivery effectively inhibited MDR1 expression at both mRNA and protein levels and decreased P-glycoprotein function. Silencing MDR1 led to decreased cell viability and IC_50 _of Vincristine and Dactinomycin.

**Conclusions:**

Our results demonstrated that ultrasound microbubble-mediated delivery of MDR1 siRNA was safe and effective in L2-RYC cells. MDR1 silencing led to decreased P-glycoprotein activity and drug resistance of L2-RYC cells, which may be explored as a novel approach of combined gene and chemotherapy for yolk sac carcinoma.

## Background

Yolk sac carcinoma are the most common malignant germ cell tumors in children, which are commonly found in the ovary, testes, sacrococcygeal areas and the midline of the body [1-4]. This type of germ tumors is aggressive and highly metastatic which can rapidly spread to adjoining tissues through the lymphatic system [5-7]. Meanwhile, clinical data show that yolk sac carcinoma in children have a high recurrence rate. Most of yolk sac carcinoma are refractory to chemotherapy and require a surgical resection of primary tumors and surrounding tissues including germinative glands. While surgical treatment of yolk sac carcinoma can decrease tumor recurrence to certain extent, removal of gonadal tissues may result in long-term physiological and psychological adverse effects in the affected children. Therefore, there is an urgent need to improve the chemotherapy efficacy of yolk sac carcinoma [8-10].

Tumor drug resistance is one of the most important factors which affects the outcomes of chemotherapy [11-13]. It has been well documented that certain, genes products, such as multiple drug resistance gene (MDR1), multidrug resistance-associated protein, lung resistance protein, glutathione-S-transferase Pi, contribute to drug resistance [14-17]. Our previous studies showed that MDR1 was the most and highest expressed resistance genes in tissues of yolk sac carcinoma in children. MDR1 gene, also known as ABCB1 (ATP-binding cassette, sub-family B, member 1) gene, encodes an ATP-dependent drug transporter named permeability glycoprotein (P-glycoprotein). P-glycoprotein is an energy-dependent efflux pump that exports its substrates out of the cells. Many of chemical drugs are substrates of P-glycoprotein. P-glycoprotein plays an important role in drug kinetics, including absorption, distribution, metabolism, and excretion, which limits the accumulation of drugs inside cells and results in drug resistance [18-20]. Yolk sac carcinoma have high expression of MDR1 gene [21], so we hypothesize that small interfering RNA (siRNA) mediated silencing of MDR1 expression would improve the sensitivity of yolk sac carcinoma to chemotherapy drugs.

Ultrasound microbubble-mediated delivery is a novel, nonviral, effective and safe method for delivering drugs or genes to target organs or cells [22-26]. Recent studies have shown that ultrasound microbubble-mediated delivery improves the efficacy of gene transfection and reduces the side effects of other bioactive transfection agents, such as liposome, viral vectors [27]. In this study, we constructed and characterized three effective siRNAs targeting MDR1 gene and used ultrasound microbubble-mediated gene delivery method to effectively deliver plasmid DNA into rat yolk sac carcinoma L2 (L2-RYC) cells. Our results demonstrated that the MDR1 siRNAs effectively reduced the multiple-drug resistance of L2-RYC cells. Thus, the reported approach may represent a novel and new method of combined gene silencing and chemotherapy to combat the drug resistance of yolk sac carcinoma.

## Methods

### Cell culture and chemicals

L2-RYC cells were purchased from ATCC (Manassas, VA), and were cultured in complete Dulbecco's modified Eagle's medium (DMEM) supplemented with 10% fetal bovine serum (FBS, Hyclone, Logan, Utah, USA), 100 units/ml penicillin, and 100 μg/ml streptomycin at 37°C in 5% CO_2_.

### Construction and validation of plasmids containing siRNAs targeting MDR1

The pSEB-HUS vector (Additional file 1) containing H1 and U6 dual-promoter was used to construct the eukaryotic plasmid expressing siRNA targeting MDR1 [28]. Four pairs of oligonucleotides specific for rat MDR1 coding region (Additional file 2) were designed by using Invitrogen Block-iT RNAi Designer software. After annealed *in vitro*, four double-stranded oligonucleotides cassettes with *Sfi*I cohesive ends were subcloned into the *Sfi*I sites of pSEB-HUS vector, resulting in pSEB-siMDR1 plasmids. We transfected four pSEB-siMDR1 plasmids into L2-RYC cells with Lipfectamine 2000 and detected the inhibition efficiency of each siMDR1 by quantitative real-time polymerase chain reaction (qRT-PCR), respectively. After validation, equimolar amounts of pSEB-siMDR1-1, -2 and -3 were pooled and transfected into L2-RYC cells with liposome to detect the inhibition efficiency of MDR1 by qRT-PCR.

### Quantitative real-time PCR

As described previously [29], total RNA was extracted from L2-RYC cells after 2 days transfection using TRIZol reagent (Invitrogen, Carlsbad, CA, USA) and reverse transcripted into single-strand cDNA template with random primer and a reverse transcriptase (Takara, Japan). Primers were 18-20 mers, designed by using Primer 5 program to amplify the 3'-end of rat MDR1 and glyceraldehyde-3-phosphate dehydrogenase (GAPDH) genes (Additional file 2). Quantitative RT-PCR reaction was performed as follows: 3 min at 94°C (one cycle), 20 sec at 94°C, 20 sec at 58°C, 20 sec at 72°C, and reading plate (38 cycles). Raw data of Ct value for MDR1 in each group was normalized with GAPDH and measured as the fold change.

### Preparation of the siMDR1-loaded lipid microbubble

To prepare lipid microbubble, we mixed 5 mg of dipalmitoyl phosphatidylcholine (Sigma, USA), 2 mg of distearoyl phosphatidyl ethanolamine (Sigma, USA), 1 mg of diphenyl phosphoryl azide (Sigma, USA), and 50 μl of glycerol into phosphate buffered saline (PBS) to make the 0.5 ml mixture in a tube. The tube was placed at 40°C for 30 min, then filled with perfluoropropane gas (C3F8) and mechanically shaken for 45 sec in a dental amalgamator (YJT Medical Apparatuses and Instruments, Shanghai, China). The pure lipid microbubble was PBS diluted, sterilized by Co_60 _and stored at -20°C. Then, the home-made lipid microbubble were mixed with poly-L-lysine (Sigma, USA), and incubated at 37°C for 30 min. Subnatant was removed and washed twice by PBS. Plasmids containing balance mixed siMDR1 plasmids were added and incubated at 37°C for 30 min, and washed by PBS twice. This procedure was repeated three times. The siMDR1-loaded lipid microbubble were obtained with an average diameter of 2.82 ± 0.76 μm, an average concentration of 8.74 × 10^9^/ml and the average potential of -4.76 ± 0.82 mV (n = 5). The final concentration of plasmids DNA was 0.5 μg/μl.

### Trypan blue staining

Cultured L2-RYC cells in 6-well plates were processed with acoustic intensity of 0.25 W/cm^2^, 0.5 W/cm^2^, 0.75 W/cm^2 ^and 1 W/cm^2 ^and irradiation time of 30 sec and 60 sec, respectively. Cells were washed, trypsinized and resuspended with PBS with 10^6 ^cells per milliliter. An equal volume of 0.2% trypan blue was added to a cell suspension. Then, cell suspensions were incubated at room temperature for 3 min and loaded into a hemocytometer. With an optical microscope examination, survival cells excluding trypan blue were counted in three separate fields. Survival rate = (number of survival cells/number of total cells) × 100%.

### Transfection efficiency detected by flow cytometry

L2-RYC cells were seeded in each well of 24-well culture plates with 5 × 10^5 ^cell density and cultured in complete DMEM medium for 24 hrs before transfection. Then cells were treated with pSEB-siMDR1 pooled plasmids alone (group I), plasmids with ultrasound (group II), siMDR1-loaded lipid microbubble (group III), siMDR1-loaded lipid microbubble with ultrasound (group IV) and non-plasmid control (group V), respectively. We also set up a lipofection group (Lipo) for comparison of transfection efficiency. Cells in group II and IV were exposed to ultrasound with the radiation frequency of 1 MHz, pulse wave, sound intensity of 0.5 W/cm^2 ^for 30 sec using an ultrasound treatment meter (Institute of Ultrasound Imaging, Chongqing Medical University). Since pSEB-siMDR1 plasmids express green fluorescent protein (GFP), transfected cells were collected and suspended in 1 ml of PBS/BSA buffer at 24 hrs after transfection for flow cytometry as a measurement of transfection efficiency.

### Western blot analysis

Total proteins of L2-RYC cells in each group were extracted by using protein extraction kit (Beyotime, China, at 48 hrs after transfection. Approximately 20 micrograms total proteins per lane were loaded onto a 6% SDS-PAGE gel. After electrophoretic separation, proteins were transferred to an Immobilon-P membrane. The membrane was blocked with 5% fat-free skim milk in Tris buffered saline with tween-20 buffer at room temperature for 1 hr, and was incubated with anti-MDR1 or anti-β-actin primary antibody (Santa Cruz Biotechnology, USA), respective, at 4°C overnight. After being washed, the membrance was incubated with a secondary antibody conjugated with horseradish peroxidase (HRP) (Santa Cruz Biotechnology, USA) at room temperature for 1 hr, followed by extensive wash. The protein of interest was visualized and imaged under the Syngene GBox Image Station by using Luminata Crescendo Western HRP Substrate (Millipore, USA). The expression level of MDR1 proteins was calculated using GBox Image Tools and normalized by β-actin levels.

### Daunorubicin accumulation assay

Daunorubicin accumulation assay was conducted to determine P-glycoprotein activity [30]. L2-RYC cells were treated as above mentioned in each groups, as well as a blank control. Cells were washed and changed with FBS-free DMEM. Daunorubicin was administered into culture medium at the final concentration of 7.5 μg/ml and the cells were incubated at 37°C for 30 min. Cells were then washed with FBS-free DMEM medium again, followed by incubation with Verapamil (Pharmacia Co., Italy) at the final concentration of 10 μg/ml to end the efflux function of P-glycoprotein. Subsequently, cells were washed three times with PBS and the Daunorubicin accumulation was examined under a fluorescence microscope and analyzed by flow cytometry. (FACS Calibur FCM, Becton-Dickinson, San Jose, CA)

### MTT assay

L2-RYC cells in each treated group were seeded into 96-well culture plates with 5 × 10^3 ^cell density. After incubation in complete DMEM medium for 24 hrs, the medium was replaced with FBS-free DMEM containing Vincristine or Dactinomycin at the concentration ranges of 0.1, 0.2, 0.4, 0.8, 1.6, 3.2, 6.4, 12.8 μg/ml (for Vincristine) and 0.01, 0.02, 0.04, 0.08, 0.16, 0.32, 0.64, 1.28 μg/ml (for Dactinomycin), respectively. MTT assay was performed at 12 hrs post treatment to determine cell proliferation. Briefly, 20 μl of MTT reagent was added to each well with FBS-free DMEM medium and incubated at 37°C for 4 hrs. Medium was gently aspirated and replaced by 200 μl of DMSO. The 96-well plates were shaken for 10 min to dissolve the purple crystals and read at 520 nm in Thermo Scientific Varioskan Flash Spectral Scanning Multimode Reader. Viability of L2-RYC cells in each concentration was calculated as OD_treated_/OD_untreated _× 100%. The half maximal inhibitory concentration (IC_50_) was accounted to compare the drug sensitivity among each group.

### Statistical analyses

All data were shown as mean ± standard deviation (SD). Statistical analyses were performed using SPSS 15.0 software package (SPSS, Inc, Chicago, IL). Mann-Whitney *U *test was performed to compare results among experimental groups. *P *< 0.05 was considered as statistically significant.

## Results

### Construction and silencing efficiency of pSEB-siMDR1 plasmids expressing siRNAs against MDR1

**Figure 1:**
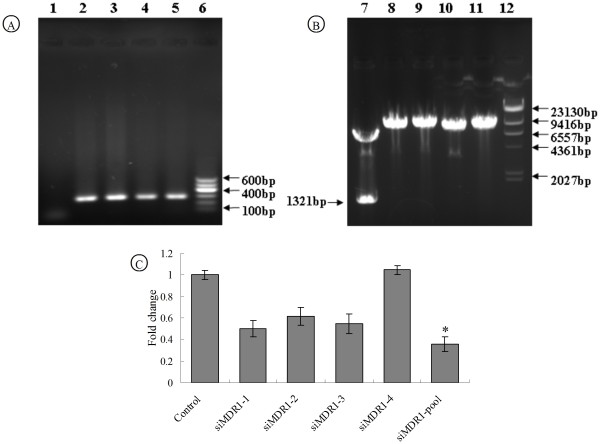
**Construction of recombined plasmids containing siMDR1 and inhibition of endogenous MDR1 gene expression**. (A) Identification of recombinant pSEB-siMDR1 plasmids by PCR amplification, About 300 bp of DNA fragment was PCR amplified from pSEB-siMDR1 plasmid template by U6 promoter primer and antisense of siRNA sequence. (1. negative control; 2. PCR product from pSEB-siMDR1-1 plasmid; 3. PCR product from pSEB-siMDR1-2 plasmid; 4. PCR product from pSEB-siMDR1-3 plasmid; 5. PCR product from pSEB-siMDR1-4 plasmid; 6. DNA Ladder, 600 bp, 500 bp, 400 bp, 300 bp, 200 bp, 100 bp). (B) Identification of recombinant pSEB-siMDR1 plasmids by *Not*I restriction enzyme digestion, No small DNA fragment was digested from corrected recombinant pSEB-siMDR1 plasmids by *Not*I enzyme compared with pSEB-HUS vehicle vector (7. *Not*Ienzyme-digested pSEB-HUS vehicle vecter; 8. *Not*Ienzyme-digested pSEB-siMDR1-1 plasmid; 9. *Not*Ienzyme-digested pSEB-siMDR1-2 plasmid; 10. *Not*Ienzyme-digested pSEB-siMDR1-3 plasmid; 11. *Not*Ienzyme-digested pSEB-siMDR1-4 plasmid;12. λ/HindIII DNA Ladder, 23130 bp, 9416 bp, 6557 bp, 4361 bp, 2322 bp, 2027 bp, 564 bp, 125 bp), (C) Silencing efficiency of MDR1 expression by siMDR1, Expression of MDR1 in L2-RYC cells with pSEB-siMDR1 plasmids lipofection for 24 hr was detected by real-time PCR. Results were normalized by GAPDH and confirmed in at least three batches of independent experiments. (*P < 0.05, vs other four single siMDR1 transfection groups and control group).

We subcloned four pairs of siRNA oligonucleotide cassettes that target rat MDR1 coding region using the previously developed pSOS system [28]. After inserting the cassettes into the pSEB-HUS vector, we were able to amplify and confirm an approximately 300 bp of PCR product in the four recombinant pSEB-siMDR1 plasmids using U6 promoter primer and antisense oligonucleotide of siRNA cassettes (Figure 1A). A *Not*I restriction enzyme site was removed when siRNA oligonucleotide cassettes were inserted into multi cloning sites of pSEB-HUS vector. When we used *Not*I to digest pSEB-siMDR1 plasmids, no about 1300 bp DNA fragment was seen in corrected recombinants compared with pSEB-HUS vector which could be cut out to be about 1300 bp DNA fragment and another large DNA fragment (Figure 1B). Next, we tested the silencing efficiency of different siRNA target sites and found that three of the four pSEB-siMDR1 plasmids transfection decreased the mRNA level of MDR1 in L2-RYC cells. The highest silencing efficiency was observed in the pooled plasmids group (Figure 1C). Therefore, for the following experiment, we chose to use the pooled plasmids to transfect cells.

### Cell survival in different ultrasound parameters

The survival rate of L2-RYC cells in different ultrasound intensities and exposure time was determined by trypan blue staining. Cell survival was more than 95% when the ultrasound parameters were set as 1 KHz, 0.25 W/cm^2 ^or 0.5 W/cm^2^, 30 sec and pulse wave. Cell death increased significantly when cell were exposed to ultrasound at the intensity of 0.75 W/cm^2 ^and 1.0 W/cm^2^. At 0.5 W/cm^2 ^acoustic intensity, survival rate were 95.22 ± 1.26% and 70.16 ± 3.49% with 30 sec and 60 sec exposure time, respectively. Nonetheless, our results indicated that ultrasound exposure within a suitable range would not affect cell survival (Table 1).

**Table 1:** Cell Viability with different ultrasound intensities and exposure time

Intensity (W/cm^2^)	Survival rate (%)	
	
	30 s	60 s
0.25	97.07 ± 1.14	96.03 ± 1.51
0.5	95.22 ± 1.26	70.16 ± 3.49
0.75	71.25 ± 3.22	51.75 ± 4.02
1	37.43 ± 3.41	23.98 ± 3.24

### Transfection efficiency and silencing efficiency of different transfection groups

**Figure 2:**
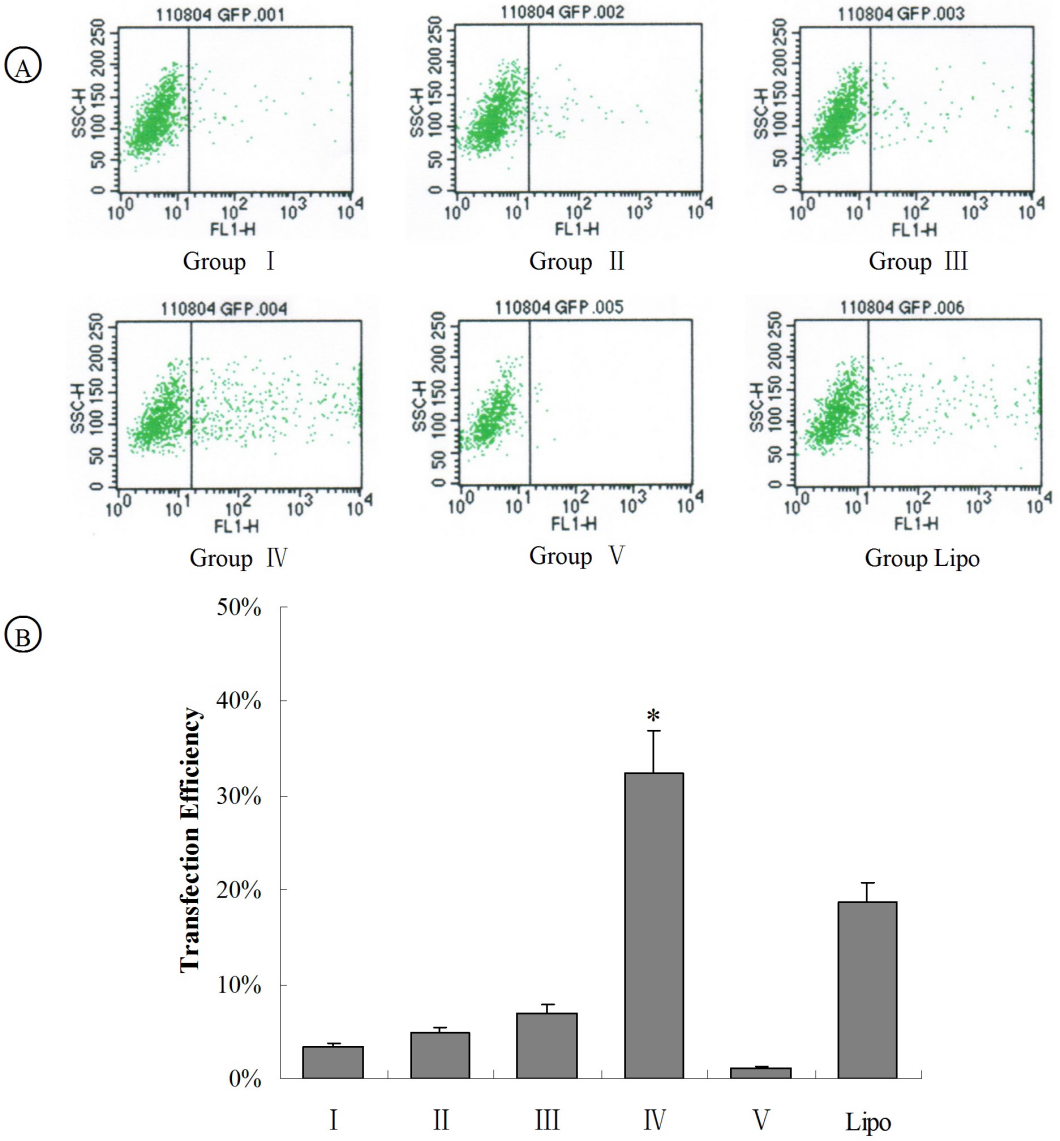
**Ultrasound-mediated siMDR1-loaded lipid microbubble increase transfection efficiency**. (A) Flow cytometry was performed to detect GFP positive cells. L2-RYC cells were treated by plasmids alone (group I), plasmids with ultrasound (group II), siMDR1-loaded lipid microbubble (group III), and siMDR1-loaded lipid microbubble with ultrasound (group IV). Untreated L2-RYC cells were used as control group (group IV), and liposome transfected L2-RYC cells were used as experimental control (group Lipo). (B) The percentage of green fluorescent cells of each group was demonstrated in a histogram. (*P < 0.05, vs other groups).

Retroviral vector pSEB-HUS contains enhanced GFP code region driven by human EF1α promoter (hEF1). Thus, GFP expression can reflect the transfection efficiency. Flow cytometry results showed that group I, II, III and IV exhibited very low transfection efficiency (< 8%) and had no significant difference among these groups. However, approximately 30% of GFP-positive cells were obtained in group IV (Figure 2A and 2B) which was significantly higher than other experimental groups, including the lipofection group (*P *< 0.05).

**Figure 3:**
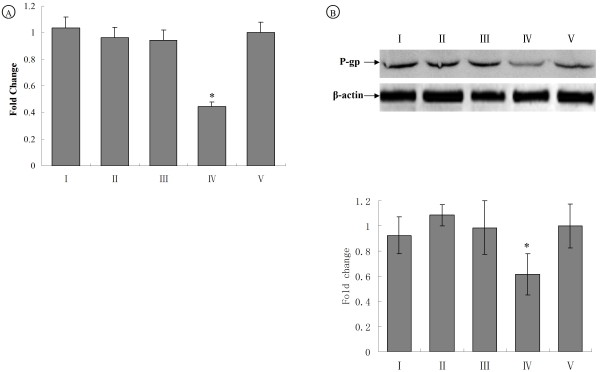
**Transfected siMDR1 inhibits the mRNA and protein expression of MDR1 in L2-RYC cells**. (A) mRNA expression of MDR1 in group I, II, III, IV and IV was analyzed by real-time PCR. All cDNA samples were normalized with GAPDH. Real-time PCR results were confirmed in at least three batches of independent experiments. (*p < 0.05, vs other groups), (B) Protein expression of MDR1 was analyzed by Western blot. Protein were collected and lysed at 48 hr after treatment and subjected to SDS-PAGE and Western blotting using a MDR1 antibody. Equal loading of the samples was confirmed by β-actin detection. All samples gray values were normalized with β-actin. P-glycoprotein protein relative expression of each group was demonstrated as fold change in a histogram. (*P < 0.05, vs other groups).

The mRNA and protein expression of MDR1 were effectively inhibited in group IV L2-RYC cells. MDR1 expression in other three groups did not decrease when compared with non-plasmid control. There was no significant difference in the mRNA and protein expression of MDR1 among group I, II, III and IV (Figure 3A and 3B). These results demonstrated that siMDR1-loaded microbubble combined with ultrasound-induced burst significantly improved transfection efficiency of plasmid and selected siRNA pool targeting MDR1 could effectively inhibit the MDR1 expression.

### Analysis of P-glycoprotein activity with Daunorubicin accumulation assay

**Figure 4:**
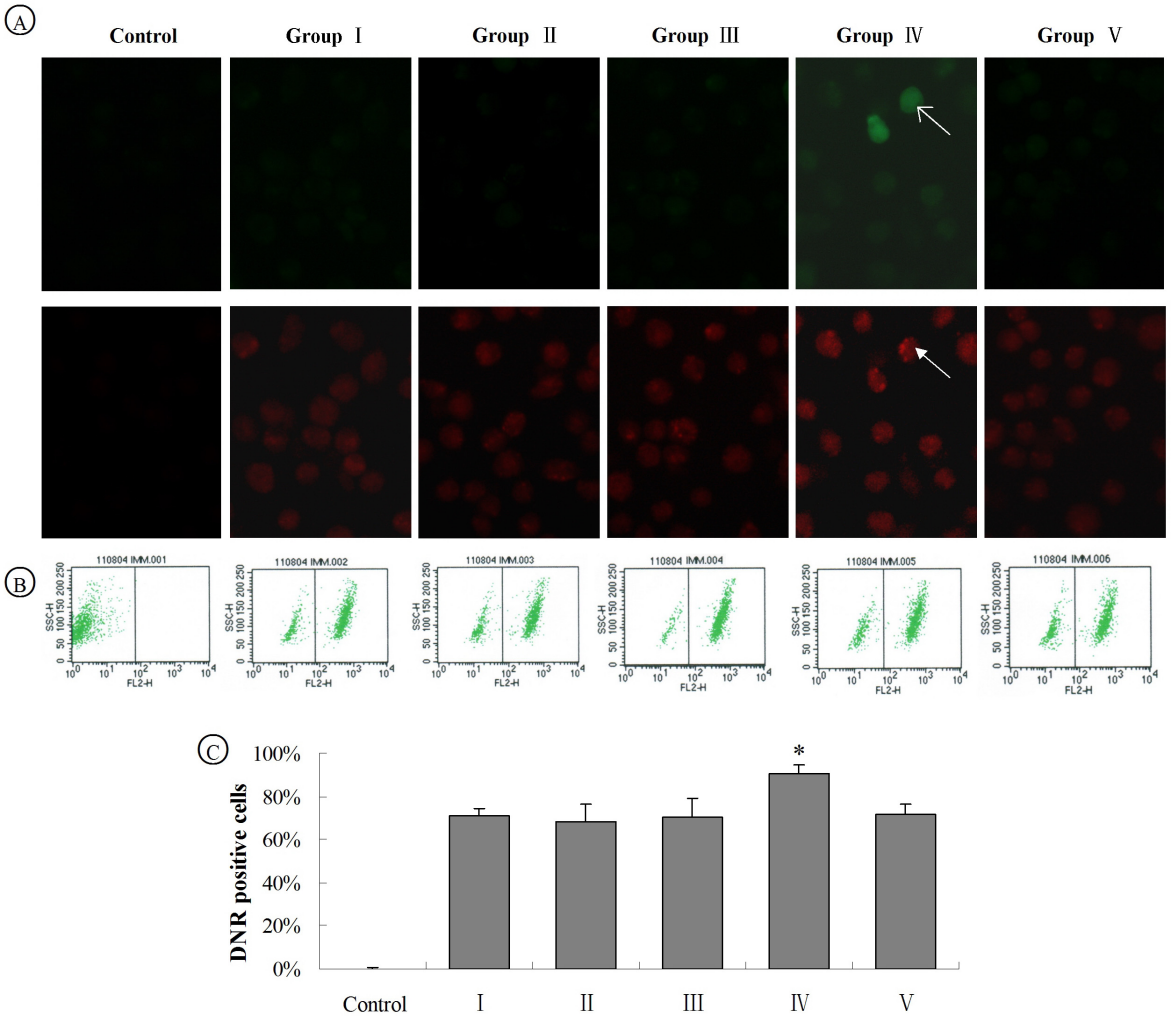
**Daunorubicin accumulation increases in the cells treated with siMDR1-loaded Lipid microbubble transfection**. The experimental groups I to V were same as that described in figure 2. L2-RYC cells were seeded in 6-well plates. Daunorubicin was added to the final concentration of 7.5 μg/ml. After 30 min, Verapamil at the final concentration of 10 μg/ml was added to terminate pumping-out of Daunorubicin. L2-RYC cells without any treatment were set as negative control. (A) Red fluorescent cells was observed under microscope, cells in group IV (cells transfected with pSEB-siMDR1s showed green fluorescent indicated by white arrow with thin arrowhead) exhibited more red granular fluorescence in cytoplasm(indicated by white arrow), (B) Red fluorescent cells were sorted by flow cytometry, (C) The percentage of red fluorescent cells of different treated groups was displayed in a histogram. (*P < 0.05, vs other groups).

Daunorubicin is a substrate of P-glycoprotein, which has red autofluorescence. Daunorubicin accumulation assay is commonly used to determine the P-glycoprotein activity [31]. We found that only cells in group IV exhibited green fluorescence and had more visible red granular fluorescence in cytoplasm when compared with cells in other groups (Figure 4A). From flow cytometry data (Figure 4B and 4C), we found that red fluorescent intensity in group I, II, III and V were 70.85%, 68.42%, 70.57% and 71.72%, respectively. On the contrary, 90.85% red fluorescent positive cells were observed in group IV. Thus, our result demonstrated that siMDR1 transfected by ultrasound microbubble-mediated delivery could inhibit P-glycoprotein function and increased intracellular accumulation of Daunorubicin in L2-RYC cells.

### Sensitivity to chemotherapeutic drugs by MTT assay

**Figure 5:**
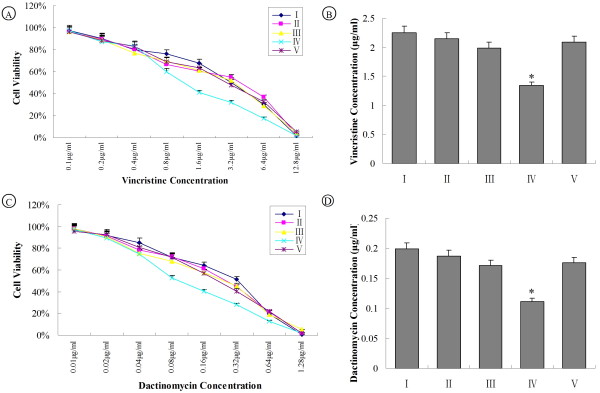
**Ultrasound microbubble-mediated siMDR1 delivery enhances the sensitivity of L2-RYC cells to chemotherapeutic drugs**. Experimental groups I to V were same as that described in figure 2. Treated cells were replanted into 96-well plates. Chemotherapeutic drugs were added into the culture at different concentrations. MTT assay was performed, and then plates were read at 520 nm by spectrophotometer. Sensitivity to chemotherapeutic drugs was determined by using cell viability and IC_50 _value. (A) Cell viability of each experimental group at different concentrations of Vincristine, (B) IC_50 _value for Vincristine in each group. (*P < 0.05, vs other groups), (C) Cell viability of each experimental group at different concentrations of Dactinomycin, (D) IC_50 _value for Dactinomycin in each group. (*P < 0.05, vs other groups)

Next, MTT assay was also performed to determine cell viability of L2-RYC cells *in vitro*. Vincristine and Dactinomycin are two commonly used chemotherapeutic drugs and also substrates of P-glycoprotein. Increased concentrations of two drugs caused reduced cell viability. Cell viability at different concentrations of two drugs and IC_50 _values were not significantly different among group I, II, III and V **(**Figure 5A and 5C). The IC_50 _of Vincristine and Dactinomycin were 1.34 μg/ml and 0.11 μg/ml in group IV which were statistically different from other groups (*P *< 0.05) (Figure 5B and 5D). Taken together, our result demonstrated that MDR1 siRNAs were transfected by ultrasound microbubble-mediated delivery could at least partially reverse drug resistance of L2-RYC cells.

## Discussion

Yolk sac carcinoma is a malignant germ cell tumor with aggressive nature in children [5,32]. While chemotherapy is critical to control the metastasis and recurrence of this disease [33], it has been reported that MDR1 expression level is related to the treatment responsiveness and prognosis in chemotherapy of malignant tumors as higher expression of MDR1 maybe lead to the lower efficiency of anti-cancer chemotherapy [20,34]. The multi-drug resistance gene MDR1 encodes an ATP-dependent efflux transporter, P-glycoprotein protein, which protects tissues or cells from environmental toxins and xenobiotics, and prevents tissues or cells from attack of anti-cancer drugs [35-37]. In this study, we investigated whether the down-regulation of MDR1 could enhance the drug sensitivity of yolk sac carcinoma *in vitro*.

Small interfering RNAs (siRNAs) mediated RNA interference is widely used to silence gene expression via transcript degradation in mammalian cells. We chose to use the pSEB-HUS system which was specific for constructing GFP vector containing siRNA. The expression of siRNA can be driven by dual convergent H1 and U6 promoters and GFP-positive cells post plasmid transfection were easily detected by flow cytometry. Any siRNA can also regulate the expression of unintended targets which have similar silent site of target gene and result in non-specific gene silence. This so-called off-target effect can not only disturb the effect of silence of RNAi but also induce toxic phenotype [38,39]. The pooling strategy of multiple target sites has been used to maximize target-gene specificity and efficiency and to minimize non-specific effects [40,41]. In this study, we first identified three effective MDR1 siRNAs from four candidate siRNA sites by qRT-PCR. The three siRNA plasmids were pooled at an equal molar concentrations and transfected into L2-RYC cells. All three siRNAs were specific for MDR1 target gene but at different mRNA degradation sites, so increased the target gene knock-down efficiency of random-designed siRNAs. The decreased concentration of individual siRNAs could reduce potential off-target effects. Our result confirmed that the pooled siRNAs have higher inhibition efficacy than that of potent individual siRNAs.

Effective siRNA DNA delivery into cells and *in vivo *has been a great challenge for the broad use of RNAi therapeutics. The most commonly used carriers for delivering nucleic acids into mammalian cells are non-viral and viral vectors. Liposome-mediated transfection is simple and powerful, but has cytotoxic side effects [26]. Calcium phosphate co-precipitation has rigorous conditions of transfection and a small range of target cells [42,43]. Virus-mediated transfection is high efficient and available to achieve sustainable transgene expression. However the biosafety for *in vivo *use remains a concern [44]. Recently, ultrasound contrast agents (in a form of microbubble) have been used to deliver gene and drug *in vitro *and *in vivo*, providing a new and efficient therapeutic technique [22-25]. Ultrasound microbubble-mediated destruction has been shown to enhance cell membrane permeability and improve gene and drug delivery. It has been shown that ultrasound microbubble-mediated destruction can transfect DNA into a variety of mammalian cells [22,24,26,45]. The change of cell membrane permeability is recoverable when ultrasound energy and exposure time are within a suitable range. Thus ultrasound exposure will not cause permanent damage to cells [45,46]. We first determined the optimal ultrasound parameters of acoustic intensity and exposure time for L2-RYC cell transfection. When cultured L2-RYC cells were exposed to ultrasound with intensity of 0.75 W/cm^2 ^and 1 W/cm^2^, the survival rates was too low to be used in the study. Although ultrasound with intensity of 0.25 W/cm^2 ^did not affect cell viability, plasmids DNA delivery into cells was poor. Fortunately, we found out ultrasound with intensity of 0.5 W/cm^2 ^for 30 s could effectively transfect plasmids into cells without causing significant amount of cell death. Our previous study on bone marrow mononuclear cells also reported gene delivery by ultrasound with intensity of 0.5 W/cm^2 ^did not reduce cell viability and not destroy membrane of treated cells [45]. Under the chosen condition, we found that 30% GFP-positive cells can be achieved by gene transfection using ultrasound microbubble-mediated delivery. This transfection was higher than that of lipofection group and significantly decreased the expression of MDR1 by more than 60%, suggesting that ultrasound microbubble-mediated delivery may be used as an effective gene delivery method.

We determined the effect of silencing MDR1 expression by ultrasound microbubble-mediated siRNA delivery on multidrug resistance of yolk sac carcinamo cells. P-glycoprotein encoded by MDR1 gene is in charge of decreasing drug accumulation in multidrug-resistant cells, including tumor cells. Daunorubicin is used in cancer chemotherapy and its subcellular distribution is related to multidrug resistance. Daunorubicin produces red fluorescence with laser excitation at 488 nm, which is readily detected in drug-treated tissues or cells. Thus, Daunorubicin accumulation assay was performed to detect P-glycoprotein activity. Our results indicated that ultrasound microbubble-mediated delivery effectively transferred siMDR1 into L2-RYC cells and led to an increased Daunorubicin accumulation.

Chemotherapeutic drugs are means to combat cancers clinically. However, drug-resistance of tumor cells severely limits therapeutic outcomes. Drug sensitivity can be estimated by tumor cell viability treated with anti-cancer drug. Vincristine and Dactinomycin both of which are most commonly used chemo drugs and also known as substrates of P-glycoprotein. Thus, MTT assay was carried out to detect cell viability at different concentrations of Vincristine and Dactinomycin and to determine the IC_50 _ratios of two drugs in each group. Our results revealed that the L2-RYC cells treated with ultrasound microbubble-mediated siMDR1 delivery became more sensitive to anti-cancer drugs. Conceivably, silencing MDR1 should achieve excellent therapeutic efficacy at lower drug dosages so that chemotherapy-associated side effects can be alleviated to certain extends.

## Conclusions

In this study, we constructed plasmids expressing siMDR1 and confirmed their silencing efficiency in L2-RYC cells. Ultrasound microbubble-mediated delivery can effectively transfer siMDR1 into L2-RYC cells and lead to inhibition of MDR1 expression and function of P-glycoprotein. Drug sensitivity was also improved by silencing MDR1. Thus, ultrasound microbubble-mediated delivery approach is a safe and effective gene transfection method and targeted inhibition method. Our results strongly suggested that combined gene silencing and chemotherapy may be further explored as a novel and potentially efficacious treatment of yolk sac carcinoma.

## Abbreviations

L2-RYC: rat yolk sac carcinoma L2 cells; MDR1: multiple drug resistance gene; P-glycoprotein: permeability glycoprotein; siRNA: small interfering RNA; DMEM: Dulbecco's modified Eagle's medium; FBS: fetal bovine serum; qRT-PCR: quantitative real-time Polymerase Chain Reaction; GAPDH: glyceraldehyde-3-phosphate dehydrogenase; GFP: green fluorescent protein; PBS: phosphate buffered saline; HRP: horseradish peroxidase; IC_50_: half maximal inhibitory concentration

## Competing interests

The authors declare that they have no competing interests.

## Authors' contributions

YH and YB carried out the experiments and drafted the manuscript; DL and SW participated in cell culture; ML and QW participated in flow cytometry; YH and JZ executed statistical analyses; ZW instructed the ultrasound technology; TL, DH, XL and GW designed the project and drafted the manuscript. All authors read and approved the final manuscript.

## Supplementary Material

Additional file 1 **Supplementary Figure 1**. Map of pSEB-HUS vector and schematic diagram of recombination. Click here for file

Additional file 2 **Supplemental table 1**. siRNA targeting MDR1 and PCR primer oligonucleotide sequence. Click here for file
