# Supplementary figures and images for: Ultrasound microbubble-mediated delivery of the siRNAs targeting MDR1 reduces drug resistance of yolk sac carcinoma L2 cells
Source: J Exp Clin Cancer Res. 2011 Oct 28;30(1):104. doi: 10.1186/1756-9966-30-104 (PMC3213040; doi:10.1186/1756-9966-30-104)

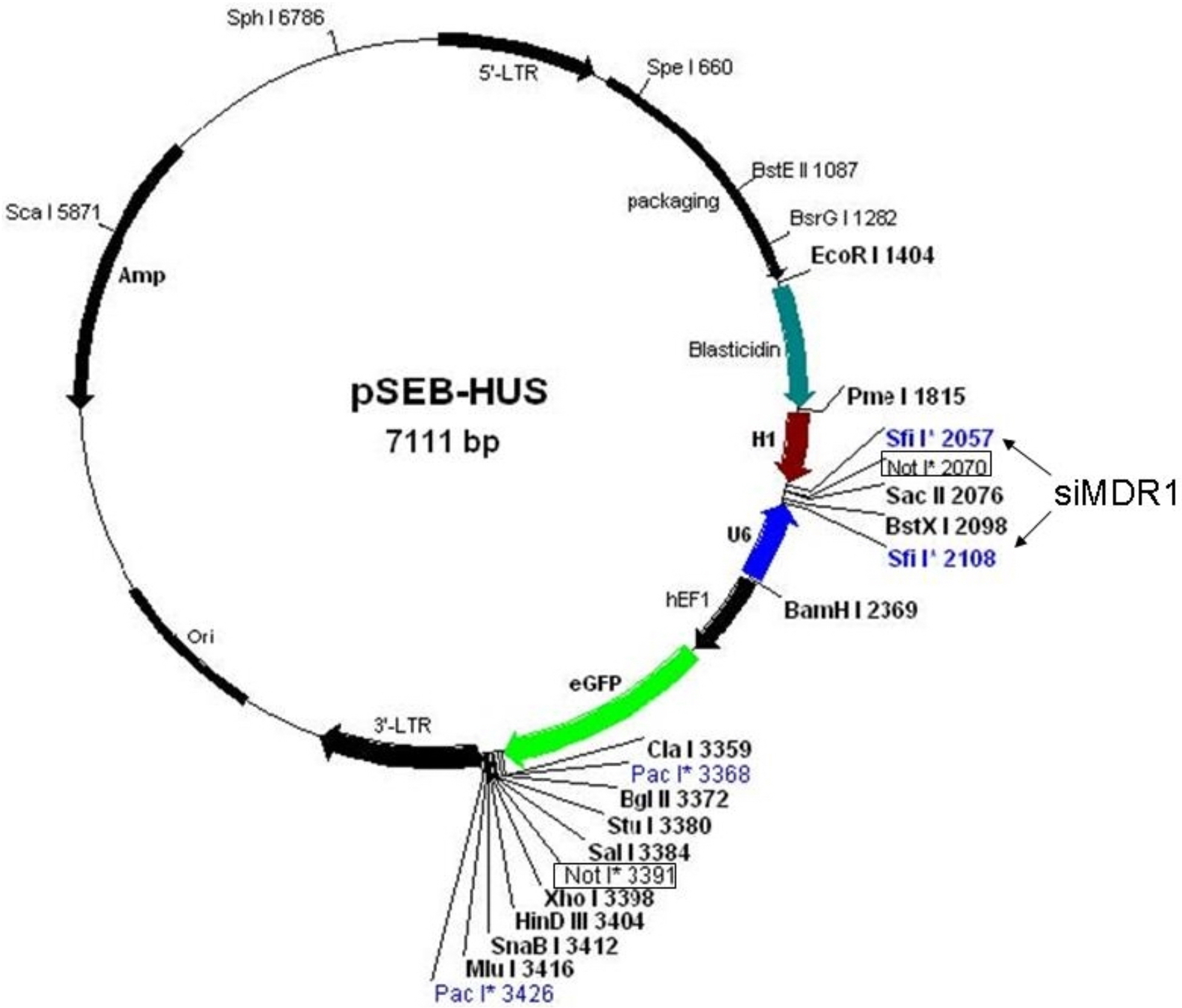

Supplement: Additional file 1 — Supplementary Figure 1. Map of pSEB-HUS vector and schematic diagram of recombination. [file 1756-9966-30-104-S1.JPEG]
