# Supplementary material for: Ultrasound microbubble-mediated delivery of the siRNAs targeting MDR1 reduces drug resistance of yolk sac carcinoma L2 cells
Source: J Exp Clin Cancer Res. 2011 Oct 28;30(1):104. doi: 10.1186/1756-9966-30-104 (PMC3213040; doi:10.1186/1756-9966-30-104)
Supplement: Additional file 2 — Supplemental table 1. siRNA targeting MDR1 and PCR primer oligonucleotide sequence. [file 1756-9966-30-104-S2.DOC]

***He Y, et al, Supplemental Table 1***

siRNA targeting MDR1 and PCR primer Oligonucleotide sequence

| Name | Sequence |
| --- | --- |
| rat MDR1 siRNA site-1 top | aGCCGAGCGTTACTAATCAAtttt |
| rat MDR1 siRNA site-1 bottom | aTTGATTAGTAACGCTCGGCtttt |
| rat MDR1 siRNA site-2 top | aGGTCCTCTCAAATGAATATtttt |
| rat MDR1 siRNA site-2 bottom | aATATTCATTTGAGAGGACCtttt |
| rat MDR1 siRNA site-3 top | aGAGAAGACTTAGTTCGAAAtttt |
| rat MDR1 siRNA site-3 bottom | aTTTCGAACTAAGTCTTCTCtttt |
| rat MDR1 siRNA site-4 top | aGGATATAAGCTGGTTTGATtttt |
| rat MDR1 siRNA site-4 bottom | aATCAAACCAGCTTATATCCtttt |
| rat MDR1 Fwd | GAGAACATCGCCTACGG |
| rat MDR1 Rev | GCTTCCTGGACGACCTT |
| rat GAPDH Fwd | TGGAGTCTACTGGCGTCTT |
| rat GAPDH Rev | GCTGACAATCTTGAGGGAG |
| U6 promotor Fwd | CGCGGATCCAAGGTCGGGCAGGAAGAG |
